# The association of polypharmacy and high-risk drug classes with adverse health outcomes in the Scottish population with type 1 diabetes

**DOI:** 10.1007/s00125-021-05394-7

**Published:** 2021-02-19

**Authors:** Andreas Höhn, Anita Jeyam, Thomas M. Caparrotta, Stuart J. McGurnaghan, Joseph E. O’Reilly, Luke A. K. Blackbourn, Rory J. McCrimmon, Graham P. Leese, John A. McKnight, Brian Kennon, Robert S. Lindsay, Naveed Sattar, Sarah H. Wild, Paul M. McKeigue, Helen M. Colhoun, on behalf of the Scottish Diabetes Research Network (SDRN) Epidemiology Group

**Affiliations:** 1 MRC Institute of Genetic and Molecular Medicine, University of Edinburgh, Edinburgh, UK; 2 Division of Molecular and Clinical Medicine, University of Dundee, Dundee, UK; 3 Ninewells Hospital, Dundee, UK; 4 Western General Hospital, NHS Lothian, Edinburgh, UK; 5 Queen Elizabeth University Hospital, Glasgow, UK; 6 Institute of Cardiovascular and Medical Sciences, University of Glasgow, Glasgow, UK; 7 Usher Institute of Population Health Sciences and Informatics, Centre for Population Health Sciences, School of Molecular, Genetic and Population Health Sciences, University of Edinburgh, Edinburgh, UK; 8 Public Health, NHS Fife, Kirkcaldy, UK

**Keywords:** Acute complications of diabetes, Ageing, DKA, High-risk prescribing, Hypoglycaemia, Medication reviews, Mortality, Multimorbidity, Polypharmacy, Type 1 diabetes

## Abstract

**Aims/hypothesis:**

The aim of this work was to map the number of prescribed drugs over age, sex and area-based socioeconomic deprivation, and to examine the association between the number of drugs and particular high-risk drug classes with adverse health outcomes among a national cohort of individuals with type 1 diabetes.

**Methods:**

Utilising linked healthcare records from the population-based diabetes register of Scotland, we identified 28,245 individuals with a diagnosis of type 1 diabetes on 1 January 2017. For this population, we obtained information on health status, predominantly reflecting diabetes-related complications, and information on the total number of drugs and particular high-risk drug classes prescribed. We then studied the association of these baseline-level features with hospital admissions for falls, diabetic ketoacidosis (DKA), and hypoglycaemia or death within the subsequent year using multivariate Cox proportional hazards models.

**Results:**

Not considering insulin and treatment for hypoglycaemia, the mean number of prescribed drugs was 4.00 (SD 4.35). The proportion of individuals being prescribed five or more drugs at baseline consistently increased with age (proportion [95% CI]: 0–19 years 2.04% [1.60, 2.49]; 40–49 years 28.50% [27.08, 29.93]; 80+ years 76.04% [67.73, 84.84]). Controlling for age, sex, area-based socioeconomic deprivation and health status, each additional drug at baseline was associated with an increase in the hazard for hospitalisation for falls, hypoglycaemia and death but not for DKA admissions (HR [95% CI]: falls 1.03 [1.01, 1.06]; DKA 1.01 [1.00, 1.03]; hypoglycaemia 1.05 [1.02, 1.07]; death 1.04 [1.02, 1.06]). We found a number of drug classes to be associated with an increased hazard of one or more of these adverse health outcomes, including antithrombotic/anticoagulant agents, corticosteroids, opioids, antiepileptics, antipsychotics, hypnotics and sedatives, and antidepressants.

**Conclusions:**

Polypharmacy is common among the Scottish population with type 1 diabetes and is strongly patterned by sociodemographic factors. The number of prescribed drugs and the prescription of particular high-risk drug classes are strong markers of an increased risk of adverse health outcomes, including acute complications of diabetes.

**Graphical abstract:**

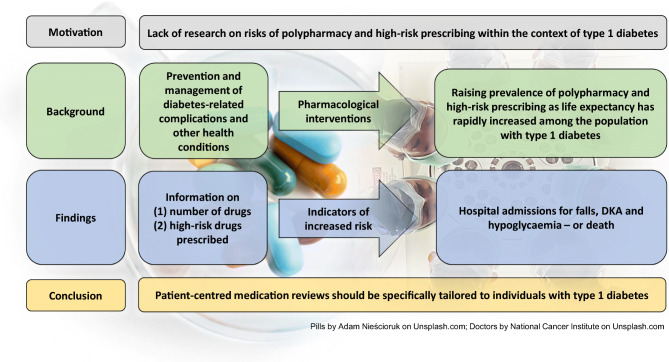

**Supplementary Information:**

The online version of this article (10.1007/s00125-021-05394-7) contains peer-reviewed but unedited supplementary material.

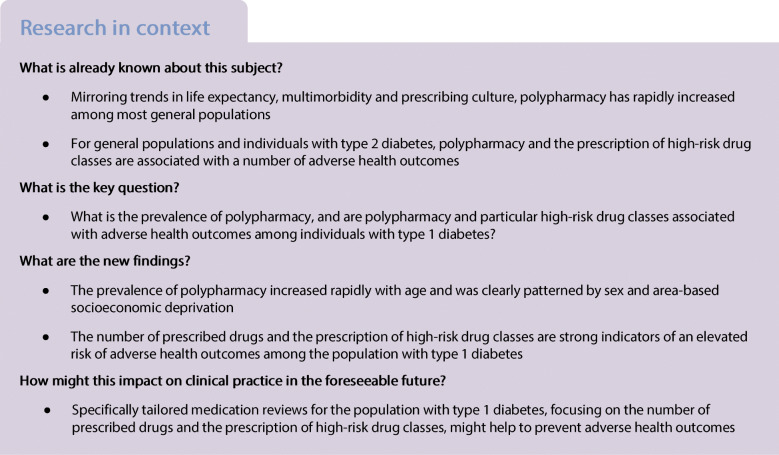



## Introduction

Life expectancy has rapidly increased among general populations and among the population with type 1 diabetes [1]. Due to improvements in the prevention and treatment of diabetes-related complications, more individuals with type 1 diabetes are living longer [2, 3]. However, the incidence of most diabetes-related complications and non-communicable diseases increases with age [4, 5]. The fact that more individuals with type 1 diabetes are reaching older ages is therefore accompanied by several challenges associated with multimorbidity (the presence of two or more long-standing chronic conditions) [6]. Two of these challenges are the increasing prevalence of polypharmacy, often defined numerically as being exposed to five or more drugs with potential for drug–drug interactions [7], and the increasing number of individuals being exposed to drugs that are associated with an increased risk of medication errors and adverse drug reactions [8].

For general populations, the prevalence and risks of polypharmacy and high-risk prescribing are relatively well explored [9, 10]. Polypharmacy and the prescription of high-risk drugs were shown to be strongly associated with an increased risk of adverse health outcomes [11–13]. However, identifying the extent to which these risks reflect direct drug effects, drug–drug interactions or the health conditions for which drugs are prescribed is difficult in observational studies [14]. Despite the complexity of disentangling direct drug effects from confounding by indication, the potential harm from polypharmacy and high-risk prescribing has led to clinical recommendations to minimise the potential risks in Scotland and the UK [15, 16].

For particular disease groups and subpopulations, including individuals with type 2 diabetes, the prevalence of polypharmacy and its association with adverse health outcomes is increasingly recognised [17–20]. In contrast, very little is known for the population with type 1 diabetes. This is surprising, as the management of type 1 diabetes and its complications often require early pharmacological interventions, implying that individuals are often exposed to complex medication regimes for a long period of time [21, 22].

Using data for the entire Scottish population with type 1 diabetes, we mapped the number of prescribed drugs over age, by sex and area-based socioeconomic deprivation on 1 January 2017. We then studied the association of each additional drug and the prescription of particular high-risk drugs at baseline with the first hospital admission for falls, diabetic ketoacidosis (DKA) and hypoglycaemia, or death within the subsequent 12 months. All studied outcomes represent important endpoints: hospital admissions for DKA and hypoglycaemia are among the major acute complications of type 1 diabetes; falls and death are among the most reported complications of polypharmacy among general populations. In line with findings for general populations and individuals with type 2 diabetes, we expected the number of prescribed drugs among individuals with type 1 diabetes to increase with age, to be higher among the female sex than the male sex, and to be higher among individuals from more deprived areas. Furthermore, we hypothesised that each additional drug and the prescription of high-risk drug classes would be associated with an increased hazard for hospitalisation for falls, DKA and hypoglycaemia as well as death. These findings will provide important evidence to improve appropriate prescribing among individuals with type 1 diabetes.

## Methods

### Data sources

We utilised pseudonymised, population-based data, extracted from the Scottish Care Information-Diabetes (SCI-Diabetes) collaboration database, which is a comprehensive register of all individuals assigned a diagnosis of diabetes in primary or secondary care in Scotland. In Scotland, healthcare in the National Health Service (NHS) is free at the point of delivery, providing a strong incentive to use national screening programmes. The register captures more than 99% of all diabetes cases in Scotland, covering clinical data and information on prescriptions in primary care [23]. A routinely applied algorithm, based on age, drug prescriptions and clinical information on the type of diabetes, was used to identify individuals with type 1 diabetes in the SCI-Diabetes database [23]. Using the Community Health Index (CHI), a unique personal identification number, records were linked with information on hospital admissions from the Scottish Morbidity Record 01 (SMR01) dataset provided by the Information Services Division (ISD) of the NHS in Scotland. In addition, we were able to link these data with information on the date and the cause of death, provided by National Records of Scotland (NRS).

### Ethics approval

Data and data linkage were set up with approval from the Scottish A Research Ethics Committee (ref 11/AL/0225), Caldicott Guardians and the Privacy Advisory Committee (PAC - reference 33/11), now running with approval from the Public Benefit and Privacy Panel for Health and Social Care (PBPP - reference 1617-0147).

### Study population

Using the SCI-Diabetes database, we identified all individuals resident in Scotland, irrespective of their age, who were alive and had a diagnosis of type 1 diabetes at baseline, defined as 1 January 2017 (*N* = 28,245). For each individual, we counted the total number of prescribed drugs at baseline. Identical chemical substances, identified using the seventh digit of the Anatomical Therapeutic Chemical Classification System (ATC) of the WHO, were counted as one drug [24]. Individuals with type 1 diabetes require insulin, which is often prescribed together with drugs to manage hypoglycaemia. Insulins, glycogenolytic hormones and carbohydrates were not considered when counting the number of prescribed drugs. In addition, we did not count devices such as insulin pumps, flash monitors or needles.

We also examined whether individuals were prescribed high-risk drugs. We decided to focus on those second- and third-level ATC classes that were consistently reported to be strongly associated with an increased risk of serious medication errors and adverse drug reactions, leading to hospital admissions, disabilities or death [8, 25, 26]. While the use of such drugs can of course be clinically appropriate, it is recommended that these drug classes are critically reviewed periodically by the handling practitioner according to the recent Scottish Polypharmacy and Appropriate Prescribing Guidelines [15]. From a total number of 4747 unique ATC codes, 769 (16.20%) unique ATC codes were captured as high-risk drugs.

We used the Scottish Index of Multiple Deprivation (SIMD) 2016 as an area-based measure of socioeconomic deprivation. The SIMD is an area-level index that captures social deprivation across multiple aspects of life, including unemployment, income, education and crime rates at an individual’s place of residence [27].

Measures of baseline characteristics for the study population were identified within a 2 year window prior to baseline. If multiple measurements were available, the measurement closest to baseline was used. These measures were mainly recorded in primary care and included diabetes duration, HbA_1c_, systolic BP, diastolic BP, HDL-cholesterol, LDL-cholesterol, total cholesterol, BMI, diabetic foot risk score, retinopathy grading, eGFR, smoking status, and whether individuals used continuous subcutaneous insulin infusion (CSII). The diabetic foot risk score reflects the maximum score of either the left or the right foot. The retinopathy grading is based on a score combining the maximum grading of each eye. Measures of eGFR were adjusted for individuals receiving renal replacement therapy and categorised as <30 (ml min^−1^ [1.73 m]^−2^).

In addition, we obtained information on whether individuals were previously admitted to hospital for CVD, hypoglycaemia and DKA using all available information on hospital admissions. We obtained information on the number of hospital admissions within the 2 year period prior to baseline, not considering admissions for DKA and hypoglycaemia.

The daily dose of insulin at baseline was conceptualised as the mean daily dose of insulin per day within a 360 day window ranging from 180 days before baseline to 180 days after baseline. For each individual, we combined information on quantity, pack size and strength of all insulin prescriptions to estimate the total amount of issued insulin within this 360 day window. We then assumed that 20% of all prescribed insulin is not taken due to damage, loss, a passed expiry date or deviance from the established treatment regimen. While no study has quantified this ‘waste factor’ explicitly for the population with type 1 diabetes in Scotland, we followed results discussed in the literature [28]. We then divided the corrected, total amount of issued insulin by the number of days individuals were observable within this 360 day window. A small number of unrealistically high and low daily doses of insulin were identified using the 0.5% (left) and 1% (right) tails of a fitted log-normal distribution, the shape of which described the original data best.

The insulin dose for all identified outliers and all missing sociodemographic and health information at baseline were imputed using multiple imputation methods, based on all covariates presented in this study. An overview on the fraction of all imputed missing data at baseline is provided in ESM Table 1. Imputations were carried out using the R-package Amelia (R version 3.6.0, Amelia version 1.7.6; downloaded via https://cran.r-project.org/).

### Statistical analysis

We used summary statistics to describe the characteristics of the study population at baseline. In addition, summary statistics were used to map the number of drugs at baseline over age, sex and SIMD quintiles. We obtained 95% CIs for prevalence estimates using Poisson-based bootstrapping.

We followed all 28,245 individuals for a maximum period of 12 months to the end of 2017 and identified any first admission to hospital with falls, DKA and hypoglycaemia within this period as primary or secondary diagnosis. The relevant ICD-10 codes (http://apps.who.int/classifications/icd10/browse/2016/en) are shown in ESM Table 2. In addition, we identified any deaths among the study population registered in Scotland. Individuals who did not experience the studied outcome during the follow-up period were right-censored at the end of follow-up or at the point in time they became unobservable. This implied that, for all studied outcomes other than death, individuals were right-censored in case they died without being previously admitted to hospital for falls, DKA or hypoglycaemia.

We used Cox proportional hazards models to study the association between both the number of ‘each additional drug’ and the prescription of specific high-risk drugs at baseline with the first hospital admissions for falls, DKA and hypoglycaemia or death within the subsequent 12 month period. For each outcome, we modelled the effect of ‘each additional drug’ controlled for all clinical and sociodemographic covariates. For each outcome, we estimated 14 models, one for each high-risk drug class, to examine the effect of that particular drug class adjusted for clinical and sociodemographic covariates and number of additional drugs at baseline.

Regression results are presented as HRs. The δ-method was used to obtain corresponding 95% CIs. Data preparation and analyses were carried out with R (version 3.6.0).

The assumption of proportional hazards was examined using Schoenfeld residuals. Results of this analysis are shown in ESM Table 3 and indicate that the proportionality assumption was violated globally only for the model ‘Opioids–Death’. For this model, we provided the HR from the Cox model and a corresponding OR obtained from a logistic regression; these were very similar in size.

### Sensitivity analyses

We modelled the effect of ‘each additional drug’ at baseline as a linear term. However, it is possible that the effect of the number of drugs on the hazard is not linear (e.g. there could be threshold numbers of drugs above which risk increases). Therefore, in a sensitivity analysis, we examined the partial effects of the number of drugs on the hazard rate for all four outcomes. We re-ran all presented models but specified the variable ‘each additional drug’ using the most flexible form of penalised *B*-splines [29].

We investigated whether the effect of the number of drugs on the hazard rate varied over age. We estimated the effect of the number of drugs separately for the age groups 0–49 years, 50–69 years and 70+ years. In addition, we tested an interaction effect between the number of drugs and age.

## Results

### Health profile of the study population

An overview of the study population is provided in Table 1. We studied 28,245 individuals with type 1 diabetes at baseline, of which 15,731 were male (55.69%) and 12,514 were female (44.31%). The mean age was 42.31 years (SD 18.32) and the mean HbA_1c_ was 70.54 mmol/mol (SD 17.90) (8.60% [SD 3.79]). The mean diabetes duration was 20.64 years (SD 13.87). Of all studied individuals, 18.66% were previously admitted to hospital for CVD, 18.70% for hypoglycaemia and 37.56% for DKA. Among the study population, 34.95% were diagnosed with a mild or moderate retinopathy and 27.44% were diagnosed with maculopathy or pre- or proliferative retinopathy. Active ulcers or amputations were present among 7.81% of all individuals. We found that 45.79% were current or previous smokers and that 13.34% used a device for CSII therapy.

**Table 1:** Overview of individuals with type 1 diabetes in Scotland on 1 January 2017

Characteristic	Value
Male sex, *n* (%)	15,731 (55.69)
Female sex, *n* (%)	12,514 (44.31)
Age, years	42.31 ± 18.32
No. of additional drugs	4.00 ± 4.35
No. of main ATC groups	2.44 ± 2.14
Diabetes duration, years	20.64 ± 13.87
HbA_1c_, mmol/mol	70.54 ± 17.90
HbA_1c_, %	8.60 ± 3.79
Adjusted daily dose of insulin, U	62.75 ± 31.26
Systolic BP, mmHg	128.50 ± 17.09
Diastolic BP, mmHg	74.20 ± 10.42
HDL-cholesterol, mmol/l	1.53 ± 0.46
LDL-cholesterol, mmol/l	2.54 ± 0.91
Total cholesterol, mmol/l	4.66 ± 1.06
BMI, kg/m^2^	26.53 ± 5.65
No. of previous admissions excluding hypoglycaemia / DKA in the 2 years prior to baseline	1.11 ± 3.04
Previous CVD admission, *n* (%)	5271 (18.66)
Previous DKA admission, *n* (%)	10,608 (37.56)
Previous hypoglycaemia admission, *n* (%)	5283 (18.70)
CSII therapy, *n* (%)	3768 (13.34)
Ever smoker, *n* (%)	12,933 (45.79)
CKD-EPI eGFR, *n* (%)	
≥90 ml min^−1^ [1.73 m]^−2^	18,695 (66.19)
60–89 ml min^−1^ [1.73 m]^−2^	7034 (24.90)
30–59 ml min^−1^ [1.73 m]^−2^	1876 (6.64)
<30 ml min^−1^ [1.73 m]^−2^	640 (2.27)
Retinopathy / maculopathy, *n* (%)	
None	10,624 (37.61)
Mild / moderate	9871 (34.95)
Maculopathy / pre- or proliferative retinopathy	7750 (27.44)
Foot risk score, *n* (%)	
Low	19,189 (67.94)
Moderate	3802 (13.46)
High	3047 (10.79)
Active ulcer / amputation	2207 (7.81)
First-level ATC groups, *n* (%)	
ATC A: Alimentary tract and metabolism	8847 (31.32)
ATC B: Blood and blood forming organs	8147 (28.84)
ATC C: Cardiovascular system	12,742 (45.11)
ATC D: Dermatologicals	3138 (11.11)
ATC G: Genito urinary system and sex hormones	5038 (17.84)
ATC H: Systemic hormonal preparations, excluding sex hormones and insulins	4700 (16.64)
ATC J: Anti-infective for systemic use	4346 (15.39)
ATC L: Antineoplastic and immunomodulating agents	849 (3.01)
ATC M: Musculoskeletal system	2453 (8.68)
ATC N: Nervous system	10,478 (37.10)
ATC P: Antiparasitic products, insecticides, repellents	570 (2.02)
ATC R: Respiratory system	5464 (19.35)
ATC S: Sensory organs	1892 (6.70)
ATC V: Various	254 (0.90)

Data are presented as mean±SD or *n* (%)

Not considering insulin and treatment for hypoglycaemia, the mean number of prescribed drugs was 4.00 (SD 4.35). The most frequently prescribed ATC groups were cardiovascular system drugs (45.11% prevalence), nervous system drugs (37.10%) and alimentary tract and metabolism drugs (31.32%).

Table 2 provides an overview of the prevalence of all studied high-risk drug classes at baseline. Out of all the 28,245 individuals, 13,204 (46.75%) were prescribed at least one of the drugs listed in Table 2. Among those, the most frequent drug classes were antidepressants (*n* = 5384 [19.06%]), diuretics (*n* = 4462 [15.8%]) and calcium channel blockers (*n* = 2977 [10.54%]).

**Table 2:** Individuals with type 1 diabetes in Scotland on 1 January 2017 on all studied high-risk drug classes and mean number of prescribed drugs among those individuals who were prescribed this particular high-risk drug class

Drug class	Absolute number	Percentage	Mean
A10B: Blood-glucose-lowering drugs, excluding insulin	1945	6.89	1.09
B01A: Antithrombotic/anticoagulant agents	1448	5.13	1.02
C03: Diuretics	4462	15.80	1.19
C07: Beta blockers	2315	8.20	1.00
C08: Calcium channel blockers	2977	10.54	1.00
H02: Corticosteroids	917	3.25	1.89
L04A: Immunosuppressants	545	1.93	1.35
M01A: Nonsteroidal anti-inflammatory agents	1751	6.20	1.04
N02A: Opioids	1602	5.67	1.09
N03A: Antiepileptics	2250	7.97	1.11
N05A: Antipsychotics	613	2.17	1.14
N05B: Anxiolytics	620	2.20	1.03
N05C: Hypnotics and sedatives	599	2.12	1.03
N06A: Antidepressants	5384	19.06	1.14

### Prevalence of polypharmacy

**Fig. 1:**
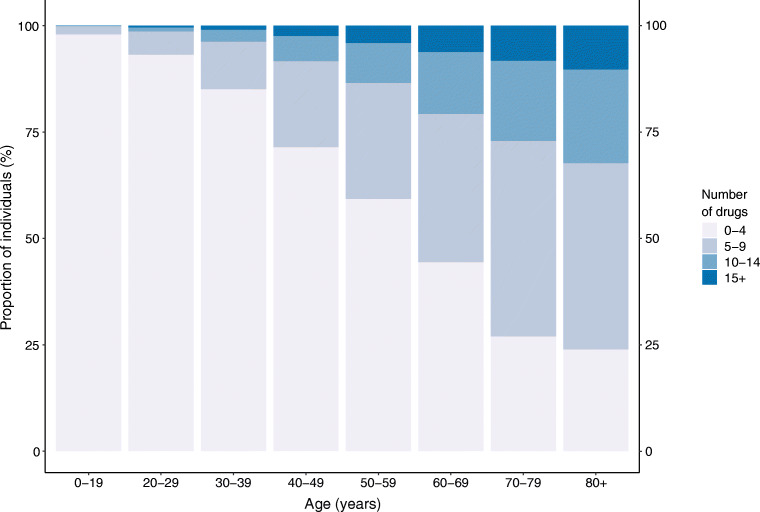
Prescribed drugs in the Scottish population with type 1 diabetes on 1 January 2017, not considering insulin and treatment for hypoglycaemia

Figure 1 illustrates the number of prescribed drugs (not considering insulin and treatment for hypoglycaemia) by age at baseline. The prevalence of individuals prescribed five or more drugs increased from 2.04% (95% CI 1.60, 2.49) among individuals aged 0–19 years to 28.50% (95% CI 27.08, 29.93) among those aged 40–49 years and 76.04% (95% CI 67.73, 84.84) among those aged 80 years and older.

A more detailed overview of prescribed medication patterns over age and by sex is given in ESM Fig. 1 and ESM Table 4. ESM Fig. 2 provides an overview on age patterns across the most frequently prescribed first-level ATC groups.

**Fig. 2:**
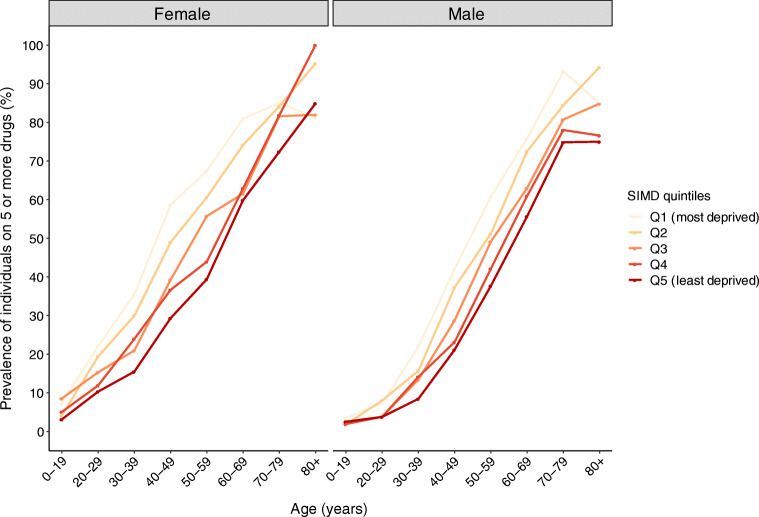
Prevalence of individuals prescribed five or more drugs in the Scottish population with type 1 diabetes on 1 January 2017 for age categories, by sex and SIMD, not considering insulin and treatment for hypoglycaemia

The number of prescribed drugs differed by sex and area-based socioeconomic deprivation. Figure 2 shows the prevalence of individuals on five or more drugs, not considering insulin and treatment for hypoglycaemia, over age and by SIMD quintile. In both sexes, the prevalence of individuals on five or more drugs was higher among individuals from more deprived areas than among those from less deprived areas; this difference was more marked in the female sex than in the male sex. As an illustration, the prevalence of individuals in SIMD quintile 5 (least deprived area) aged 40–49 years prescribed five or more drugs was 21.14% (95% CI 17.22, 25.24) among the male sex and 29.31% (95% CI 24.35, 34.52) among the female sex. The corresponding prevalence in individuals in SIMD quintile 1 (most deprived area) was 42.07% (95% CI 37.26, 47.02) among the male sex and 58.69% (95% CI 51.69, 65.68) among the female sex.

### Association with adverse health outcomes

Within the study period, we observed 308 admissions for falls, 1450 admissions for DKA and 497 admissions for hypoglycaemia. In addition, we observed 431 deaths. A detailed overview of the number of events over age is provided in ESM Table 5.

Table 3 presents results of all estimated Cox models. The number of additional drugs at baseline (not considering insulin and treatment for hypoglycaemia) was associated with a significant increase in the hazard for hospital admissions for falls, hypoglycaemia and death but not admissions for DKA (HR [95% CI]: falls 1.03 [1.01, 1.06]; DKA 1.01 [1.00, 1.03]; hypoglycaemia 1.05 [1.02, 1.07]; death 1.04 [1.02, 1.06]).

**Table 3:** HR (95% CI) derived from multivariate Cox proportional hazards models for factors associated with hospital admissions for falls, DKA, hypoglycaemia and death among people with type 1 diabetes in Scotland in 2017

Variable	Falls	DKA	Hypoglycaemia	Death
Each additional drug^a^	1.03 (1.01, 1.06)	1.01 (1.00, 1.03)	1.05 (1.02, 1.07)	1.04 (1.02, 1.06)
Drug class^b^				
A10B: Blood-glucose-lowering drugs, excluding insulin	1.00 (0.57, 1.43)	0.75 (0.46, 1.05)	0.74 (0.29, 1.18)	0.60 (0.16, 1.03)
B01A: Antithrombotic/anticoagulant agents	1.22 (0.90, 1.54)	1.26 (1.03, 1.50)	1.30 (1.00, 1.60)	0.89 (0.64, 1.14)
C03: Diuretics	1.08 (0.80, 1.36)	0.88 (0.71, 1.05)	0.88 (0.63, 1.13)	1.04 (0.81, 1.26)
C07: Beta blockers	1.04 (0.73, 1.35)	0.93 (0.71, 1.14)	1.02 (0.74, 1.31)	1.02 (0.78, 1.25)
C08: Calcium channel blockers	0.92 (0.62, 1.21)	1.09 (0.88, 1.30)	0.66 (0.36, 0.97)	0.71 (0.47, 0.95)
H02: Corticosteroids	0.89 (0.36, 1.41)	1.09 (0.82, 1.36)	1.73 (1.38, 2.07)	1.32 (0.95, 1.69)
L04A: Immunosuppressants	0.56 (0.00, 1.29)	0.76 (0.35, 1.18)	0.69 (0.14, 1.23)	0.66 (0.15, 1.16)
M01A: Nonsteroidal anti-inflammatory agents	1.13 (0.75, 1.52)	1.06 (0.85, 1.27)	0.78 (0.40, 1.16)	0.60 (0.17, 1.02)
N02A: Opioids	1.25 (0.90, 1.60)	1.12 (0.92, 1.32)	0.75 (0.42, 1.07)	1.66 (1.37, 1.95)^c^
N03A: Antiepileptics	1.29 (0.97, 1.61)	1.17 (1.00, 1.34)	1.10 (0.83, 1.36)	0.79 (0.51, 1.06)
N05A: Antipsychotics	1.22 (0.66, 1.77)	1.45 (1.21, 1.69)	1.45 (1.05, 1.85)	2.68 (2.33, 3.03)
N05B: Anxiolytics	1.49 (1.00, 1.98)	0.99 (0.70, 1.28)	1.40 (1.01, 1.79)	1.35 (0.94, 1.77)
N05C: Hypnotics and sedatives	1.61 (1.15, 2.07)	1.17 (0.90, 1.44)	1.06 (0.65, 1.47)	1.74 (1.39, 2.08)
N06A: Antidepressants	1.96 (1.70, 2.21)	1.30 (1.16, 1.43)	1.16 (0.94, 1.38)	1.26 (1.04, 1.47)

^a^Controlled for all covariates with the exception of exposure to all specific high-risk drug classes

^b^Controlled for all covariates, including the number of additional drugs at baseline, but not for the exposure to all other specific high-risk drug classes

^c^Value for Model ‘N02A: Opioids – Death’ reports OR and 95% CI from logistic regression. Corresponding value from the Cox model: 1.69 (1.42, 1.96)

Furthermore, we found several of the potentially high-risk drug classes to be associated with an increased hazard of adverse health outcomes. Antithrombotic/anticoagulant agents were significantly associated with hospital admissions for DKA (HR 1.26 [95% CI 1.03, 1.50]), while corticosteroids were associated with an increased hazard for admissions for hypoglycaemia (HR 1.73 [95% CI 1.38, 2.07]). In particular, we found nervous system drugs to be associated with an increased hazard for adverse health outcomes. For example, antidepressants were associated with hospital admissions for falls (HR 1.96 [95% CI 1.70, 2.21]), DKA (HR 1.30 [95% CI 1.16, 1.43]) and death (HR 1.26 [95% CI 1.04, 1.47]). In addition, antipsychotics were associated with an increased hazard for hospital admissions for DKA (HR 1.45 [95% CI 1.21, 1.69]), hypoglycaemia (HR 1.45 [95% CI 1.05, 1.85]) and death (HR 2.68 [95% CI 2.33, 3.03]) and anxiolytics were associated with hospital admissions for hypoglycaemia (HR 1.40 [95% CI 1.01, 1.79]).

### Results of sensitivity analyses

We assumed the effect of the number of drugs on a hazard to be linear and examined this assumption. Results of this analysis are shown in ESM Fig. 3 and suggested no departure from this.

We also investigated whether the effect of the number of drugs on the hazard for all four studied outcomes varied over age. Results of this analysis are shown in ESM Table 6 and indicate that the effect varied across age groups. As an example, while the effect of the number of drugs was not significantly associated with DKA admissions when considering all age groups, the effect was significant among the 50–69 years age group. However, the effect of the number of drugs on fall admissions was significant when all age groups were considered, while the effect was not significant among the age group 0–49 years.

Furthermore, we examined the significance of an interaction term between the number of drugs and age. We found this effect to be only significant for DKA admissions. While the main effect of the number of drugs was not significant, the direction of the interaction effect suggested that the effect of each additional drug increased the hazard rate for DKA admissions with increasing age.

## Discussion

### Principle findings

This analysis shows the high prevalence of polypharmacy and high-risk drug prescribing among the Scottish population with type 1 diabetes. Not considering insulin and treatment for hypoglycaemia, roughly one-quarter of individuals were taking five or more drugs by 40 years of age. By 60 years of age, around half of all individuals were taking five or more drugs. The levels of polypharmacy observed in this study are higher than those reported for the general Scottish population [9, 30], even when excluding insulin and drugs for the management of hypoglycaemia.

We found the prevalence of polypharmacy among individuals with type 1 diabetes to be higher among the female sex than among the male sex. Similar patterns have been reported for general populations. A higher number of prescriptions among the female sex may partly reflect that they are more likely to live longer, accumulating a larger number of chronic and disabling conditions [31]. In addition, our findings on the prevalence of polypharmacy mirror patterns observed in the general Scottish population with respect to differences by area-based socioeconomic deprivation [30, 32]. We found the frequency of individuals on five or more drugs to be substantially higher in more deprived areas than in less deprived areas. This may be explained by higher prevalence of underlying health conditions for which these drugs are prescribed, since many conditions show socioeconomic gradients, but may also in part reflect over-prescribing.

We found each additional drug at baseline to be associated with an increase in the hazard for hospitalisation for falls and hypoglycaemia, and death over 1 year follow-up. We furthermore found several potentially high-risk drug classes to be associated with an increased hazard for adverse health outcomes. Such associations may reflect risks for the conditions for which these drugs are prescribed or may be a consequence of direct drug effects. Our focus was not on differentiating between these two explanations but on highlighting the extent of polypharmacy and the potential for adverse health outcomes following from this. At the very least, polypharmacy and high-risk prescribing are important markers of elevated risk of the outcomes studied in this population, irrespective of causality.

### Comparisons with previous studies

To our knowledge, no previous study has explicitly addressed the association between polypharmacy and particular high-risk drug classes with adverse health outcomes among individuals with type 1 diabetes. One explanation for this lack of research could be that guideline recommendations typically consider polypharmacy among individuals with type 1 and type 2 diabetes unavoidable and clinically beneficial [33, 34]. Indeed, polypharmacy and the prescription of particular high-risk drug classes can be appropriate [15].

The associations with health outcomes we report echo results on the association of polypharmacy and high-risk drugs with adverse health outcomes among general populations and, with respect to polypharmacy, among individuals with type 2 diabetes [14, 17, 35–37]. These previous studies have shown that both factors, polypharmacy and high-risk prescribing, were associated with falls, incontinence, delirium, depression and insomnia, resulting in elevated hospitalisation and mortality rates.

### Strengths and limitations

In this cohort study, we used routinely collected, electronic healthcare records covering the entire Scottish population with type 1 diabetes. In contrast to survey data, these data substantially reduce the risk of selection bias and loss to follow-up. In addition, information on drug use from registers are not affected by recall bias of self- or proxy-respondents, which have a significant impact on the generalisability of findings investigating medication patterns by age, sex and socioeconomic status [38].

Findings of this study are subject to limitations. Information on prescriptions does not include information on whether drugs were taken, so we cannot directly measure medication adherence. In addition, the data only included drug prescriptions recorded in general practice and do not cover any over-the-counter drugs or drugs given in hospitals and social care facilities, possibly resulting in an underestimation of drug exposure.

Healthcare in Scotland is free and universal at point of delivery. The findings of this study might therefore not be generalisable to healthcare contexts in which access to primary care is not free of charge and purchase of drugs requires out-of-pocket expenditure.

The most important limitation is that the associations between drugs and adverse health outcomes are likely to be subject to confounding by indication. We have not attempted to construct causal models or conduct propensity score adjustments in this initial study of polypharmacy and high-risk prescribing. Such causal analyses would require more detailed drug-by-drug analysis and access to all diagnostic codes assigned in primary care that we do not currently have access to.

### Practical implications

This study highlights the prevalence of polypharmacy among individuals with type 1 diabetes, and its role as a potential marker of increased risk of adverse health outcomes, such as hypoglycaemia. Our findings should increase awareness of the potential risks that might flow from polypharmacy. Number of drugs may be a useful proxy for risk of adverse outcomes that could prompt clinicians to review drug needs periodically.

With an observational study design, it is difficult to quantify the extent to which polypharmacy-associated outcomes are directly causal or reflect confounding by indication. Direct effects of polypharmacy could arise from component single drug adverse reactions, drug–drug interaction and drug–disease interaction as well as off-target effects on the nervous system (e.g. anticholinergic or extrapyramidal effects) or cardiovascular system (e.g. hypotension). All of these associations with polypharmacy have been noted in other patient populations [39]. Another mechanism of harm from polypharmacy is that adherence to effective medications is known to fall proportional to the number of prescribed drugs [40].

People with type 1 diabetes attending many different specialist clinics may have new drugs added for specific problems without adequate consideration given to their full range of health conditions and existing medication regimens. In current guidelines, such as the ‘Realistic Prescribing’ programme in Scotland [15], patient-centred medication reviews have been proposed as the means to reduce prescribing harm in the general population [41]. It would seem prudent that people with type 1 diabetes who are on ten or more drugs, or individuals approaching the end of life, should have such reviews conducted as is recommended for the general population. Recent, in-depth qualitative studies have highlighted that in practice patient-centred reviews often do not involve the patients and do not lead to change in prescribing for complex reasons including primary care physician time and reluctance to contradict specialist prescribing [42]. Therefore the impact of patient-centred reviews on prescribing levels and health outcomes is mixed [43–45].

Our study highlights the need for a patient-centred medication review to be tailored specifically for the population with type 1 diabetes, who are at risk of a specific set of adverse outcomes. Intervention studies, aimed at improving prescribing in the context of type 1 diabetes, are needed.

### Conclusion

Our findings indicate that polypharmacy is common and strongly patterned by sociodemographic factors among the population with type 1 diabetes in Scotland. The number of prescribed drugs and the prescription of high-risk drug classes are strong risk markers for adverse health outcomes, including acute complications of diabetes.

## Supplementary Information

ESM 1 (PDF 888 kb)

## Data Availability

Data and program code cannot be made publicly available. The Scottish Diabetes Research Network can be contacted in order to gain further information on the data and legislations regarding data access (https://www.nhsresearchscotland.org.uk/research-areas/diabetes/about-the-network).

## Abbreviations

ATC: Anatomical Therapeutic Chemical Classification System
CSII: Continuous subcutaneous insulin infusion
DKA: Diabetic ketoacidosis
NHS: National Health Service
SCI-Diabetes: Scottish Care Information-Diabetes
SIMD: Scottish Index of Multiple Deprivation
