# Supplementary material for: The association of polypharmacy and high-risk drug classes with adverse health outcomes in the Scottish population with type 1 diabetes
Source: Diabetologia. 2021 Feb 19;64(6):1309–19. doi: 10.1007/s00125-021-05394-7 (PMC8099818; doi:10.1007/s00125-021-05394-7)
Supplement: Supplementary file 1 — (PDF 888 kb) [file 125_2021_5394_MOESM1_ESM.pdf]

Electronic Supplementary Material (ESM)
The Association of Polypharmacy and High-Risk Drug Classes with Adverse Health Outcomes in the Scottish Population with Type 1 Diabetes

Tables

ESM Table 1: Percentage of missing information at baseline. Note: Missing baseline-level information among the variables presented in the table were imputed using all available baseline-level information of these variables combined using multiple imputation methods.

Information	Percentage
ID	0.00
Sex	0.00
SIMD Quintiles	8.49
Age (years)	0.00
Diabetes Duration (years)	0.00
HbA1c (mmol/mol)	4.96
HDL (mmol/L)	22.49
LDL (mmol/L)	44.98
Tchol (mmol/L)	17.08
SBP (mmHG)	7.81
DBP (mmHG)	7.83
BMI (kg/m^2)	9.52
eGFR (ml/min/1.73m^2)	9.78
Retinopathy Grading	15.83
Prev. Hypoglycemia Admission	0.00
Prev. DKA Admission	0.00
Prev. CVD Admission	0.00
Smoking Status	23.92
CSII Therapy	0.00
Num. Prev. Admission Excl. Hypo./DKA (2y)	0.00
Foot Risk Score	25.65

ESM Table 2: ICD-10 codes of studied adverse health outcomes.

Condition	ICD-10 Codes
Fall	W00.-W19.
DKA	E10.0, E10.1, E11.0, E11.1, E12.0, E12.1, E13.0, E13.1, E14.0, and E14.1
Hypogylcemia	E16.0, E16.1, and E16.2

ESM Table 3: Test of proportional hazards assumption for multivariate cox models presented in Table 3. This table presents the model-specific global p-value using Schoenfeld residuals. Note: p-values > 0.05 indicate that the PH assumption is met on a global level.

Model	pVal Falls	pVal DKA	pVal Hypo.	pVal Death
Each additional drug	0.34	0.25	0.33	0.09
- Drug Class -				
A10B: Blood-glucose-lowering, excl. Insulin	0.17	0.18	0.37	0.12
B01A: Antithrombotic/ Anticoagulant agents	0.38	0.14	0.36	0.12
C03: Diuretics	0.37	0.29	0.37	0.11
C07: Beta blockers	0.38	0.29	0.37	0.10
C08: Calcium channel blockers	0.38	0.29	0.29	0.11
H02: Corticosteroids	0.31	0.29	0.36	0.11
L04A: Immunosuppressant	0.39	0.24	0.28	0.11
M01A: Nonsteroidal anti-inflammatory agents	0.34	0.29	0.30	0.11
N02A: Opioids	0.15	0.13	0.34	0.03
N03A: Anti-epileptics	0.38	0.28	0.37	0.11
N05A: Antipsychotics	0.38	0.28	0.38	0.06
N05B: Anxiolytics	0.39	0.27	0.28	0.08
N05C: Hypnotics and sedatives	0.39	0.30	0.37	0.06
N06A: Antidepressants	0.38	0.30	0.30	0.12

ESM Table 4a: Absolute number of prescribed drugs in the Scottish population with type 1 diabetes over age on 1 January 2017, not considering insulin and treatment for hypoglycemia.

Age	0-4 Drugs	5-9 Drugs	10-14 Drugs	15+ Drugs	Total
0-19	3850	77	<7	<7	NA
20-29	4173	244	43	16	4476
30-39	3851	504	128	42	4525
40-49	3820	1080	317	126	5343
50-59	3026	1391	478	206	5101
60-69	1385	1087	454	191	3117
70-79	363	617	254	110	1344
80+	98	179	90	42	409

ESM Table 4b: Absolute number of prescribed drugs among men in the Scottish population with type 1 diabetes over age on 1 January 2017, not considering insulin and treatment for hypoglycemia.

Age	0-4 Drugs	5-9 Drugs	10-14 Drugs	15+ Drugs	Total
0-19	2026	24	<7	<7	NA
20-29	2399	74	16	<7	NA
30-39	2338	234	50	15	2637
40-49	2311	552	139	46	3048
50-59	1822	770	241	86	2919
60-69	813	639	236	89	1777
70-79	172	308	112	45	637
80+	54	71	34	12	171

ESM Table 4c: Absolute number of prescribed drugs among women in the Scottish population with type 1 diabetes over age on 1 January 2017, not considering insulin and treatment for hypoglycemia.

Age	0-4 Drugs	5-9 Drugs	10-14 Drugs	15+ Drugs	Total
0-19	1824	53	<7	<7	NA
20-29	1774	170	27	13	NA
30-39	1513	270	78	27	1888
40-49	1509	528	178	80	2295
50-59	1204	621	237	120	2182
60-69	572	448	218	102	1340
70-79	191	309	142	65	707
80+	44	108	56	30	238

ESM Table 5: Number of admissions for falls, DKA, hypoglycemia, and deaths between 1 January 2017 and 31 December 2017 over age. Note: Number of deaths among the age groups 0-19 and 20-29 are reported as the sum of both age groups.

Age	Individuals	Fall Adm.	DKA Adm.	Hypo Adm.	Deaths
0-19	3930	14	302	96	*Sum 0-19 & 20-29:*
20-29	4476	18	377	56	*15 Deaths*
30-39	4525	15	211	51	27
40-49	5343	47	209	66	57
50-59	5101	70	168	75	71
60-69	3117	57	93	76	84
70-79	1344	52	62	48	101
80+	409	35	28	29	76
All	28245	308	1450	497	431

ESM Table 6: Hazard Ratio (HR) for 'Each Additional Drug' 95% Confidence Intervals (CI), derived from multivariate cox proportional hazards models - estimated stratified by age groups - for factors associated with hospital admissions for falls, DKA, hypoglycemia, and death among people with type 1 diabetes in Scotland in 2017.
Note: HR controlled for all covariates with the exception of exposure to various specific drug classes. N equals the number of individuals within this age band.

Model	HR	CI.95	Individuals
Falls: All Ages	1.03	(1.01,1.06)	28245
Falls: Ages 0-49	1.03	(0.97,1.08)	17667
Falls: Ages 50-69	1.03	(1.00,1.07)	8549
Falls: Ages 70+	1.04	(0.99,1.08)	2029
DKA: All Ages	1.01	(1.00,1.03)	28245
DKA: Ages 0-49	1.00	(0.98,1.02)	17667
DKA: Ages 50-69	1.04	(1.01,1.07)	8549
DKA: Ages 70+	1.02	(0.98,1.07)	2029
Hypoglycemia: All Ages	1.05	(1.02,1.07)	28245
Hypoglycemia: Ages 0-49	1.06	(1.03,1.10)	17667
Hypoglycemia: Ages 50-69	1.04	(1.00,1.07)	8549
Hypoglycemia: Ages 70+	1.06	(1.01,1.10)	2029
Death: All Ages	1.04	(1.02,1.06)	28245
Death: Ages 0-49	1.08	(1.03,1.12)	17667
Death: Ages 50-69	1.05	(1.02,1.08)	8549
Death: Ages 70+	1.02	(0.98,1.05)	2029

Figures

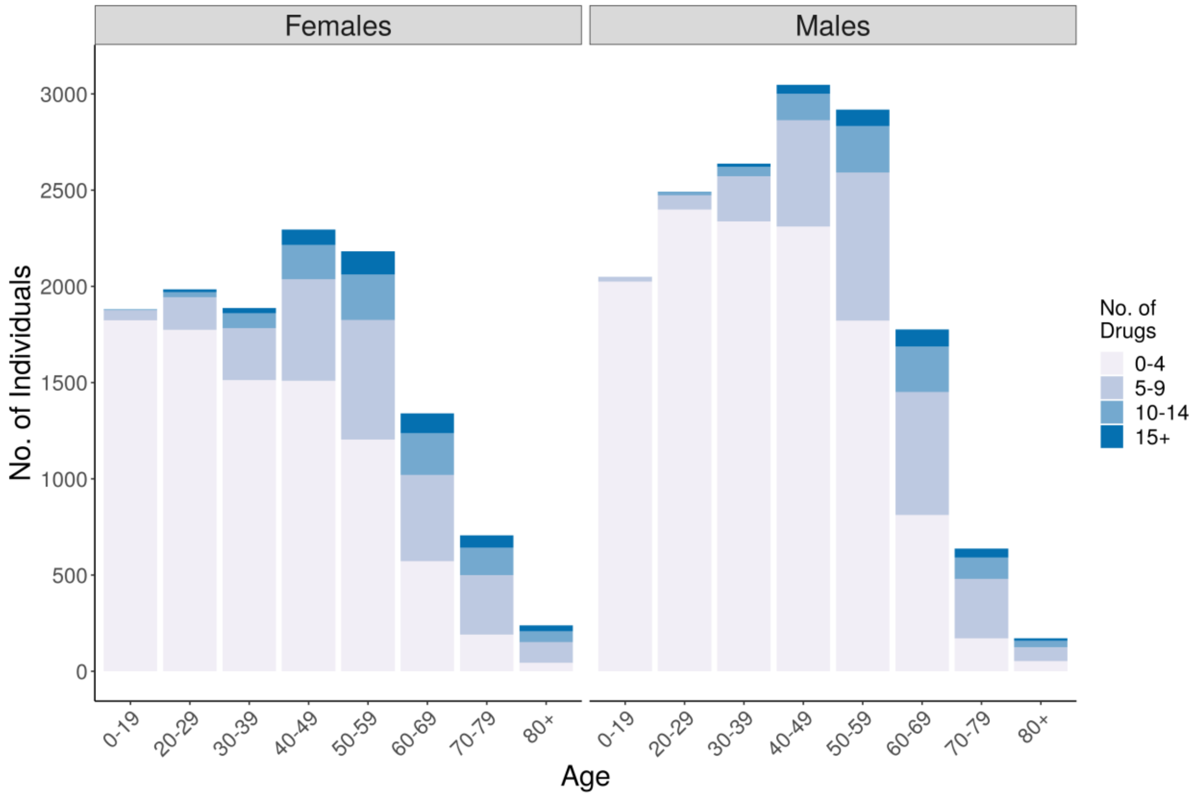

ESM Fig. 1: Absolute number of prescribed drugs in the Scottish population with type 1 diabetes over age and sex on 1 January 2017, not considering insulin and treatment for hypoglycemia.

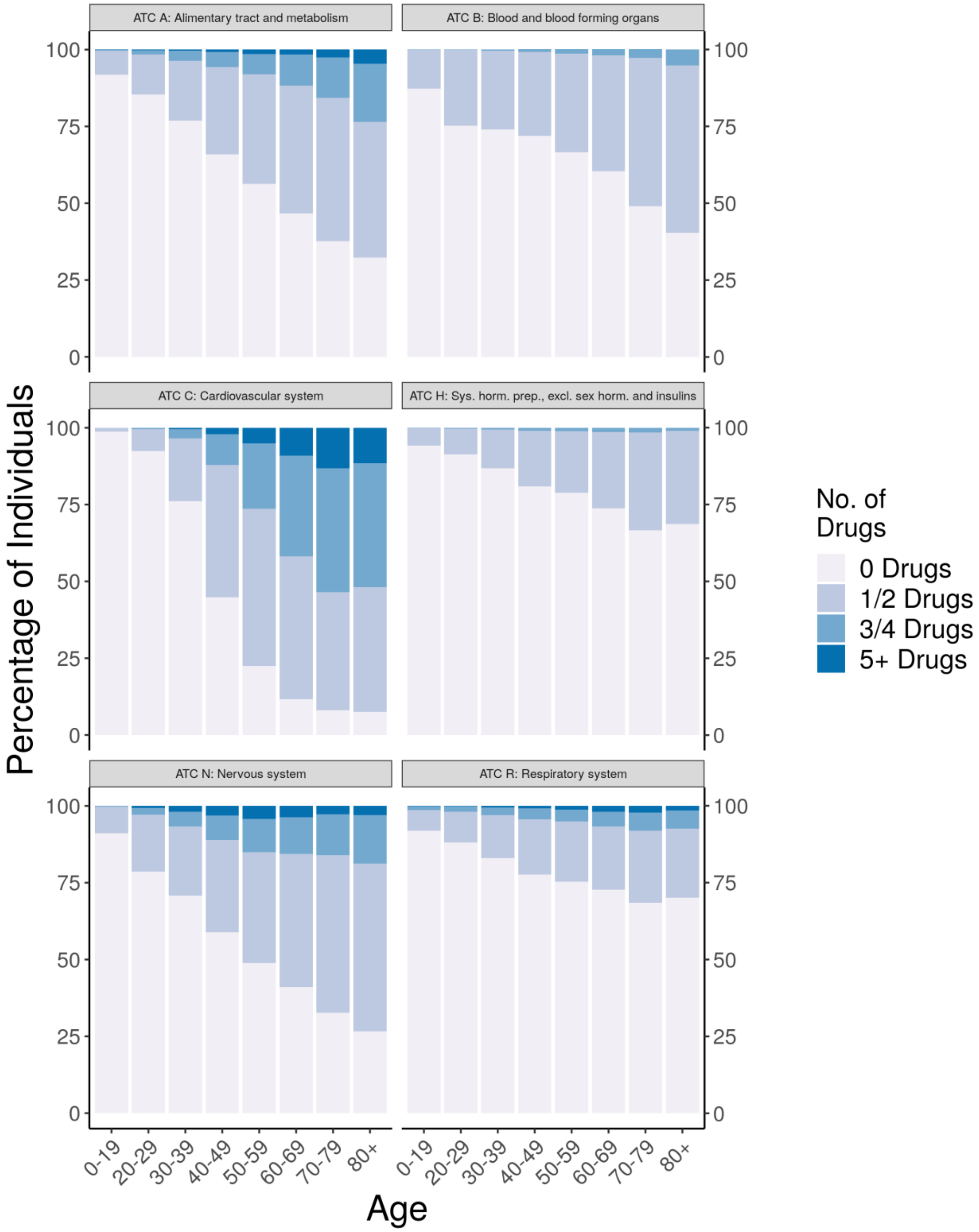

ESM Fig. 2: Prescribed drugs in the Scottish population with type 1 diabetes by Main ATC Groups on 1 January 2017, not considering insulin and treatment for hypoglycemia.

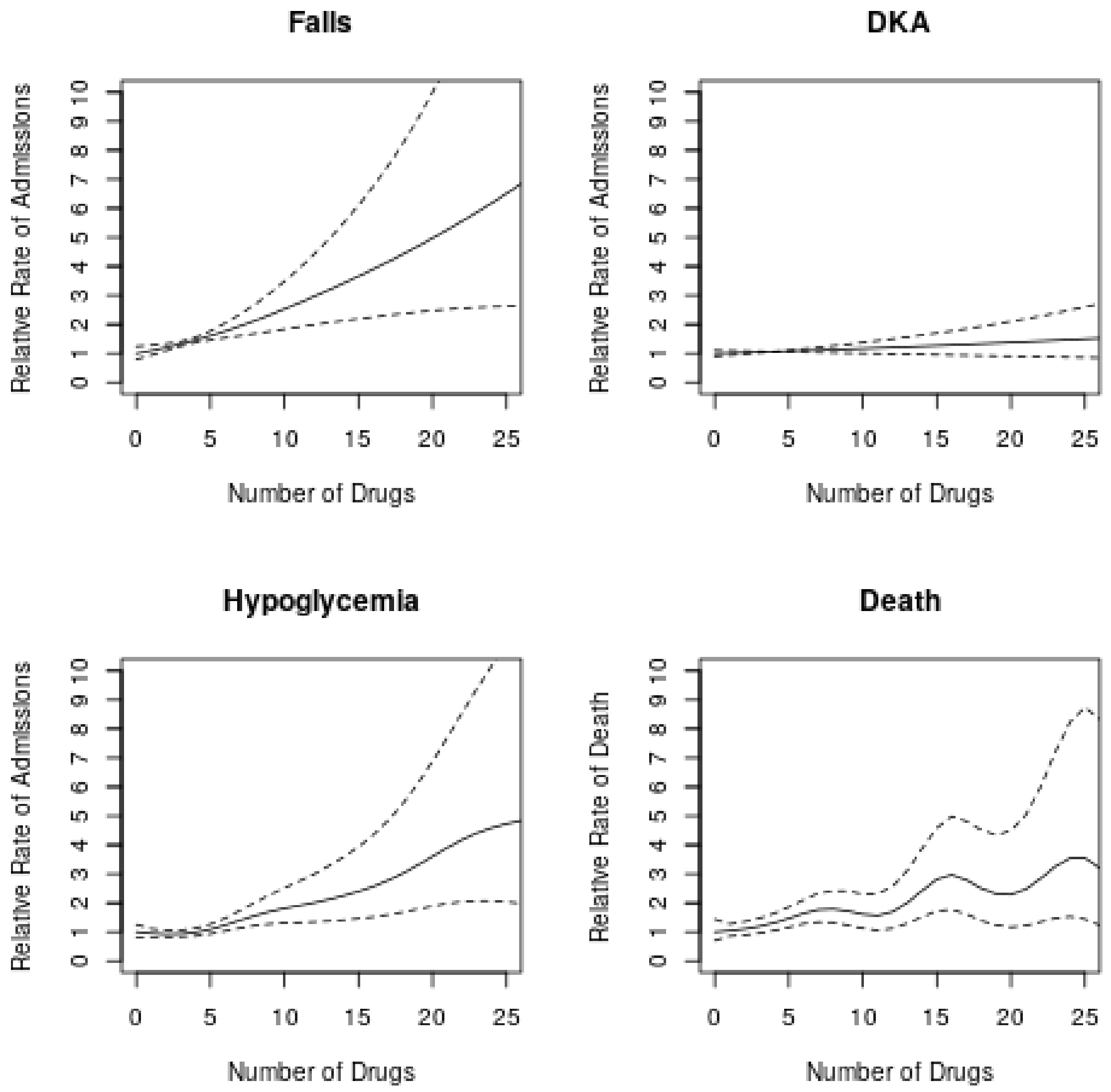

ESM Fig. 3: Partial effect of 'Each Additional Drug at Baseline' - not considering insulin and treatment for hypoglycemia - on the hazard of adverse health outcomes within the 12-month follow-up. Note: HR controlled for all covariates with the exception of exposure to various specific drug classes.
